# Classification of Complex Regional Pain Syndrome Patients Using Neuropathic Pain Symptoms—A Secondary, Retrospective Analysis From ncRNAPain

**DOI:** 10.1002/ejp.70425

**Published:** 2026-11-06

**Authors:** Bruno Rogalla von Bieberstein, Ann‐Kristin Reinhold, Frank Birklein, Cora Rebhorn, Heike L. Rittner, Gudrun Kindl

**Affiliations:** ^1^ Centre for Interdisciplinary Pain Medicine University Hospital Wuerzburg Wuerzburg Germany; ^2^ Department of Anaesthesiology, Intensive Care, Emergency Medicine, and Pain Medicine University Hospital Wuerzburg Wuerzburg Germany; ^3^ Department of Neurology University Hospital Mainz Mainz Germany

## Abstract

**Background:**

Complex Regional Pain Syndrome (CRPS) is a rare chronic pain disorder characterized by a heterogeneous symptom profile, including severe pain, allodynia and motor dysfunction. In previous work, we demonstrated elevated levels of neuropathic pain symptoms in CRPS, suggesting that refined symptom‐based stratification may improve patient classification. This study applied neuropathic pain clusters derived from the Neuropathic Pain Symptom Inventory (NPSI), which were developed by Bouhassira et al. as a phenotyping method to optimize treatment response for neuropathic pain and assessed its relevance for CRPS subgrouping.

**Methods:**

Patients (212 CRPS, 34 follow‐ups) were evaluated using standardized clinical examinations and questionnaires. They were allocated to one of three NPSI clusters (deep, evoked, pinpointed pain) at baseline and follow up after a mean interval of 2.5 years. Clustered CRPS patients were analysed for treatment differences and associations with established subgrouping methods, for example, CRPS type.

**Results:**

Most CRPS patients were allocated to the deep pain cluster, characterized by low levels of paraesthesia/dysaesthesia and pronounced pressure‐like and squeezing pain. Cluster instability was observed at follow‐up, particularly among patients initially assigned to the evoked or pinpointed pain clusters. Pinpointed pain‐clustered patients received less standard treatment.

**Conclusion:**

NPSI‐based clusters represent a promising stratification approach for CRPS that may enhance pathophysiological insights and support personalized treatment strategies.

**Significance Statement:**

This study applies the NPSI and the clusters developed by Bouhassira to classify CRPS patients based on their neuropathic symptoms. These results were compared to the complaints of the same patients after 2.5 years. Most CRPS patients remained in the deep pain cluster, even after more than 2 years. The CRPS pattern did not correlate with common classifications for CRPS.

**Trial Registration:**

German Clinical Trial Register registration number: DRKS00008964

## 1 Introduction

Complex Regional Pain Syndrome (CRPS) is characterized by persistent, disproportionate pain that typically develops following trauma or surgical intervention in middle‐aged women, most commonly in a single upper extremity (Birklein et al. 2018; Iolascon et al. 2015; Iolascon and Tarantino 2015; Castillo‐Guzmán et al. 2015).

The diagnosis of CRPS is established according to the Budapest criteria including continuing disproportionate pain, sensory, vasomotor, sudomotor/oedema and motor/trophic changes (Goebel et al. 2021; Harden et al. 2010). The clinical phenotype is highly heterogenous, leading to various proposed subclassifications based on the presence (CRPS Type II) or absence (CRPS Type I) of a nerve lesion, dystonic versus non‐dystonic presentation, inflammatory status and ‘warm’ versus ‘cold’ CRPS phenotypes (Dimova et al. 2020; Harden et al. 2010; Knudsen et al. 2023).

Although the majority of CRPS cases are classified as CRPS Type I—a form considered to be primarily nociplastic rather than neuropathic in origin—we observed notably elevated levels of neuropathic symptoms in CRPS patients reflected in the Neuropathic Pain Symptom Inventory (NPSI) in a previous study (Bouhassira et al. 2004; Dietz et al. 2019).

Using the NPSI, Bouhassira et al. (2021) developed a new classification system for neuropathic pain based on cluster analysis. This framework assigns patients with neuropathic pain to one of three clusters: (i) the deep pain cluster, characterized by pressure‐like and squeezing pain with lower levels of paraesthesia/dysaesthesia; (ii) the evoked pain cluster, defined by pain provoked elicited by brushing, cold or pressure stimuli, accompanied by lower intensities of pressure‐like pain, squeezing pain and paraesthesia/dysaesthesia; and (iii) the pinpointed pain cluster, characterized by paraesthesia or dysaesthesia and by less pain provoked by brushing, cold or pressure. Patients within the deep and evoked pain clusters responded better to treatment with subcutaneous botulinum toxin A (Bouhassira et al. 2021).

Given the pronounced clinical heterogeneity of CRPS, which has repeatedly impeded the success of clinical trials, we hypothesized that classifying neuropathic symptoms according to NPSI‐based clusters could provide a foundation for future research and offer deeper insights into underlying pathophysiological mechanisms. Based on frequently observed decreased pressure thresholds in quantitative sensory testing, we expected that most CRPS patients would cluster into the deep pain phenotype and remain within the same cluster over the course of the disease (Dietz et al. 2019). The aims of the present study were therefore threefold: first, to examine how CRPS patients distribute across the NPSI‐derived clusters; second, how they evolve over time with a follow‐up cohort; and third, to evaluate how these NPSI‐based clusters relate to currently used CRPS subgrouping approaches, like warm or cold, Type I and II CRPS.

Overall, our objective was to apply the three NPSI‐derived clusters to a CRPS cohort and to assess their potential relevance as a stratification tool.

## 2 Materials and Methods

### 2.1 Participants

Patient recruitment was conducted between 2014 and 2017 within the framework of the multicentric ncRNAPain (non‐coding RNAs) consortium after the approval of the responsible ethics committee (IRB protocol #52/14). The methodology and datasets of the ncRNAPain consortium have been described previously (Dietz et al. 2019, 2021; Dimova et al. 2020; Kindl et al. 2021; Reinhold et al. 2022). The ncRNAPain study investigated neuro‐immune alterations in the peripheral and central nervous system and their contribution to the pathophysiology of chronic pain. Exclusion criteria were malignant diseases, acute infections, autoimmune diseases and pregnancy/lactation. Of the total cohort from the three study centres of 228 CRPS patients, all 212 patients with complete NPSI data were included in this retrospective analysis. Of these 212 CRPS individuals, seventy‐four were the only members of the Würzburg cohort for whom we had contact data and who were available for follow‐up. The other individuals were followed up by the other participating study centres. Of these 74 patients, 39 were reached and approved for a non‐systematic follow‐up on average 2.5 ± 0.9 years later. Five of the 39 follow‐up patients were excluded from the final analysis due to missing NPSI data at the follow‐up visit (Figure 1).

**FIGURE 1:**
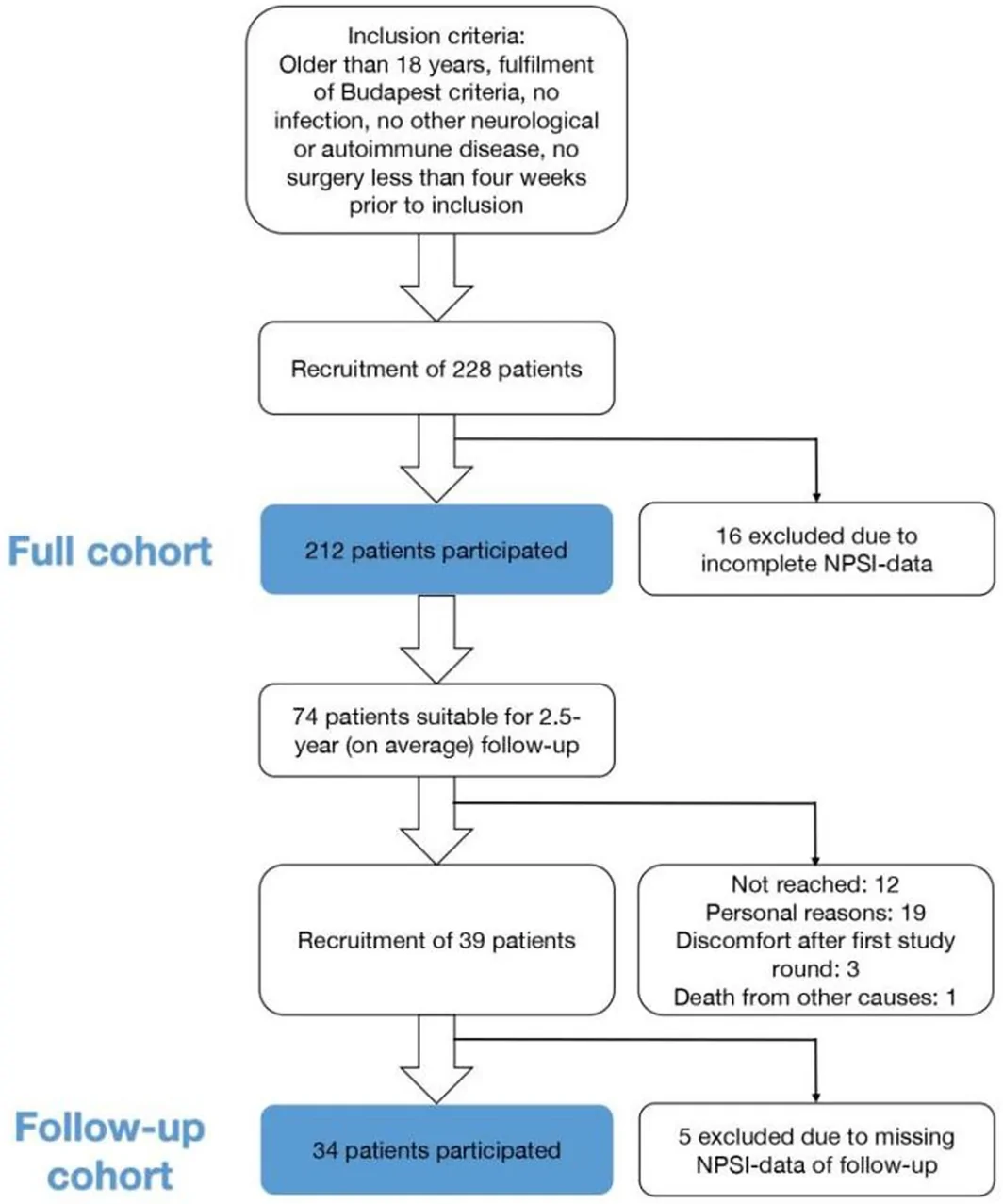
Flow‐chart of the cohorts. 212 CRPS patients of the ncRNAPain study participated at the time of study entrance (full cohort). Of these 212 patients, 74 were treated in Wuerzburg and therefore suitable for follow‐up. 34 participated in a longitudinal analysis after 2.5 (±0.9) years (follow‐up cohort).

All patients received guideline‐based treatment in accordance with the recommendations of the German Society for Neurology (DGN), following current clinical practice guidelines, including continuous physio‐ and occupational therapy as well as pain and symptom modulating medication, as described in the following sentences (Harden et al. 2022). Non‐opioid analgesics (NOPAs) consisted of either non‐steroidal anti‐inflammatory drugs (NSAIDs) or metamizole. Other analgesic medication included ketamine, baclofen, clonidine, sumatriptan, local lidocaine patches, dronabinol, vitamin c and dimethylsulfide, which were also taken for other (pain) entities. Other symptom modulating therapies consisted of bisphosphonates or steroids.

### 2.2 Clustering of Patients and Characterization of Clusters

To cluster the CRPS patients, we used the mobile version of the clustering algorithm published by Bouhassira et al. (2021) (https://juliocesar9999apps.shinyapps.io/npsi_mobile/)—for larger groups of patients the authors recommend the use of the web‐based application available at: https://npsi.shinyapps.io/NPSIclustering/ (Bouhassira et al. 2021). The clustering algorithm is based on the 10 pain‐describing items (burning, squeezing, pressure, spontaneous pain, electric shocks, stabbing, number of pain attacks, increased pain through brushing, pressure or cold) of the NPSI (Items 1–3, 5, 6 and 8–12). Individual item scores were entered into the online tool using slider controls. Upon data entry, the algorithm automatically assigned each patient to one of the predefined NPSI‐based pain clusters, based on sensory profiles and combinations of pain symptoms. The distribution of CRPS patients across clusters was analysed. At study inclusion and follow‐up, we analysed patient records and participants completed questionnaires assessing pain intensity, CRPS symptom severity (CSS), Disability of Arm, Shoulder and Hand (DASH) score, Graded Chronic Pain Scale (GCPS) classification and Quantitative Sensory Testing (QST) parameters. In addition, patients were screened for symptoms of depression using the Beck Depression Inventory (BDI) and for anxiety using the State Trait Anxiety Inventory (STA‐I). Treatment data were obtained through a systematic review of the patient records to the timepoint of study inclusion. To compare the NPSI cluster with other established subgroupings, warm/cold CRPS were defined based on the patient‐reported perceived temperature of the affected limb at disease onset. For analyses of disease duration–related effects, CRPS with a duration of more than 12 months was classified as ‘chronic’, as nearly all patients reported a pain duration exceeding 3 months at the time of study inclusion. Additionally, demographic variables including age and gender, CRPS Type 1 (without nerve injury) and 2 (with nerve injury) and the affected extremity or extremities were recorded.

### 2.3 Statistics

Normality of data distribution was assessed using the Kolmogorov–Smirnov and Shapiro–Wilk tests. Comparisons of nominal data within the CRPS cohort were performed using a Chi‐squared test. Chi‐squared tests also served to evaluate if frequency distributions of the NPSI clusters were related to other CRPS classification methods. To statistically relate nominal data—such as the proportions of patients assigned to each cluster—with distributions observed in other studies, binominal tests were applied. For longitudinal analyses (T1 vs. T0), the Wilcoxon test was used for continuous non‐parametric variables. Continuous non‐parametric data were analysed using Mann–Whitney non‐parametric tests or Kruskal–Wallis tests, as appropriate. Statistical significance was defined as *p* < 0.05.

## 3 Results

### 3.1 Prominent Deep Pain Cluster in CRPS

Both the full‐ and the follow‐up cohort were typically around 50‐year‐old females (51.2 ± 12.2 years old) and suffered from CRPS Type 1 involving a single upper extremity. At disease onset, patients most commonly reported a warm temperature sensation in the affected area. In the full cohort, approximately one third of the 212 patients reported a disease duration exceeding 1 year at the time of study inclusion. In contrast, about half of the 34 patients included in the follow‐up cohort had experienced pain for more than 1 year at baseline, and all follow‐up patients met criteria for chronic CRPS at follow‐up, conducted after a mean interval of 2.5 ± 0.9 years (Table 1).

**TABLE 1:** Demography of the cohorts: The demography of all CRPS patients in the full cohort as well as of the 34 patients in the follow‐up cohort (at both time points) is shown.

	Full cohort (T0), *n* = 212	Follow‐up cohort (T1), *n* = 34
Mean age (years)	51.15	50.83
CRPS type (% Type 1)	86.32	84.62
Primarily warm or cold CRPS (% Primarily warm)	78.61	75.00
Duration of pain at time of study entrance (% chronic/longer than 12 months)	30.29	51.52
Gender (% female)	78.30	71.43
CRPS in upper or lower extremities (% in upper)	86.19	84.62

*Note:* The age of patients was only collected at study entrance.

Abbreviations: T0, time of study entrance; T1, on average 2.5 ± 0.9 years later.

Slightly more than half of the CRPS patients were assigned to the deep pain cluster, whereas one‐third were classified within the evoked pain cluster and one sixth within the pinpointed pain cluster (Figure 2A). The NPSI Scores in the three groups compared with Kruskal–Wallis test were not significantly different (*χ* ^2^(2) = 5.258, approximate *p* = 0.072). Over the three clusters values for pain intensity, total score of the NPSI, DASH score, GCPS classification as well as depression or anxiety were numerically similar to the other clusters (Table 2). Kruskal–Wallis test showed significant differences in CSS between the pinpointed and the other two clusters (*χ* ^2^(2) = 9.681, *p* = 0.008) and in pain duration (*χ* ^2^(2) = 6.162, *p* = 0.049) between the deep pain cluster and the two remaining groups. Likewise, the clusters were not different regarding sex distribution, time since diagnosis or affected extremities (upper or lower limb) (all *χ* ^2^(df) < 2.8, ns). Notably, patients in the deep pain cluster did not exhibit reduced pressure pain thresholds in QST compared with the other clusters.

**FIGURE 2:**
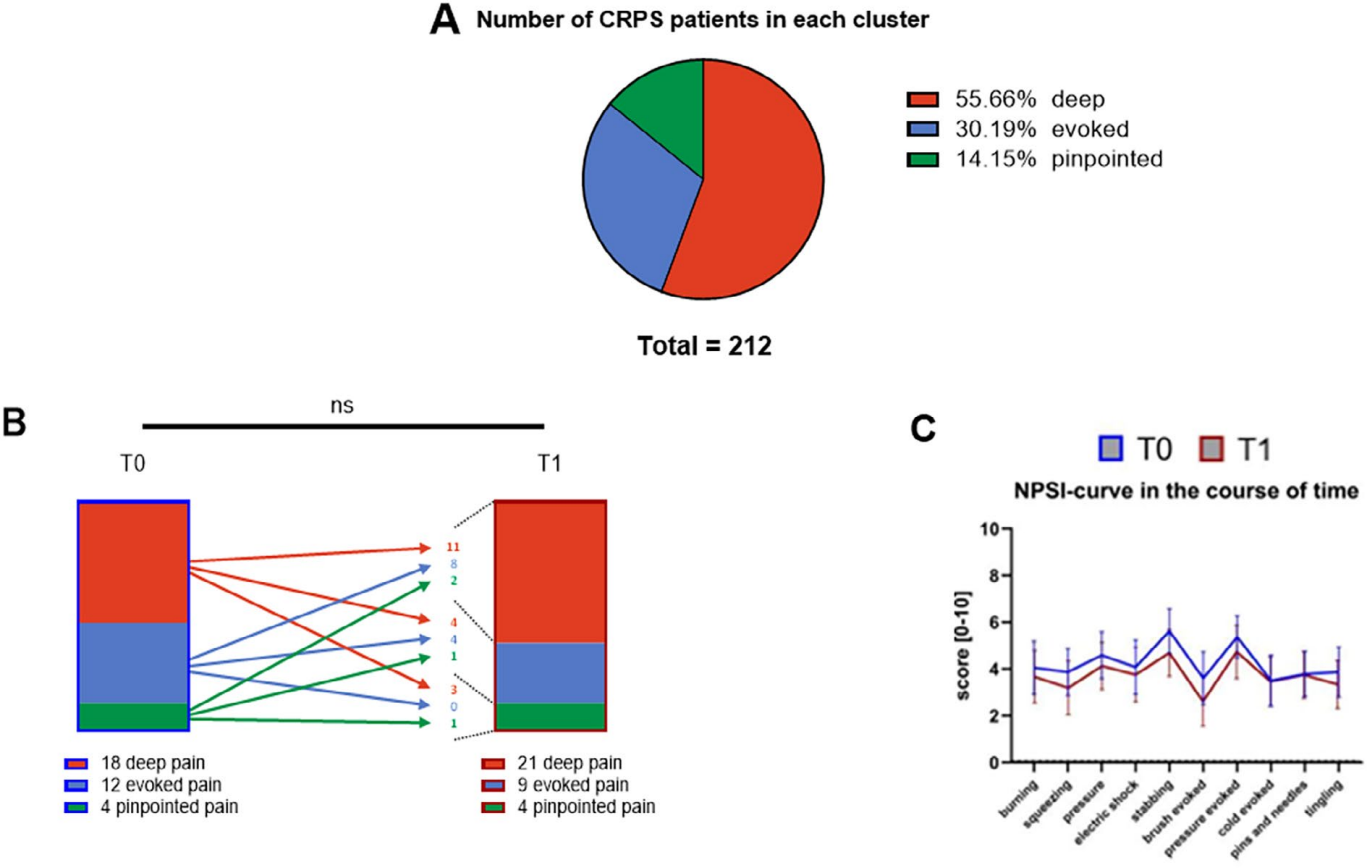
Prominent but unstable deep pain cluster in CRPS patients. (A) Using the Bouhassira algorithm (Bouhassira et al. 2021), a total of 212 CRPS patients were allocated into the three Bouhassira clusters (deep pain: *n* = 118; evoked pain: *n* = 64; pinpointed pain: *n* = 30). (B) The cluster distribution of the 34 CRPS patients of the follow‐up cohort was compared between the time of study inclusion (T0; *n* = 34) and on average 2.5 (±0.9) years later (T1; *n* = 34) using a Chi‐squared test. (C) The average scores of the pain‐describing questions of the NPSI (with 95% CI) were compared between the two time points with Wilcoxon matched‐pairs signed rank tests and were not significant (*z* = −1.035, *p* = 0.301). NPSI, Neuropathic Pain Symptom Inventory. ***p < 0.001.

**TABLE 2:** Characteristics of the three NPSI pain clusters: The different CRPS associated parameters depending on the clusters.

	Deep pain, *n* = 118	Evoked pain, *n* = 64	Pinpointed pain, *n* = 30
Pain intensity ± standard deviation	4.7 ± 2.5	5.1 ± 2.0	5.3 ± 1.7
CRPS severity score ± standard deviation	10.1 ± 2.9	11.2 ± 3.4	11.4 ± 2.3
NPSI sum score ± standard deviation	35.5 ± 25	39.4 ± 19.4	44.5 ± 19.2
Duration of pain (years) ± standard deviation	1.4 ± 2.2	1.6 ± 1.9	0.9 ± 1.5
DASH score (0–100) ± standard deviation	49.8 ± 24.3	52 ± 20.3	54 ± 18.1
GCPS score (0–4) ± standard deviation	3 ± 1.2	3.1 ± 1.1	3.4 ± 0.9
Depression BDI (%)	28.2	31.7	30
Anxiety STA‐I (%)	6.8	12.5	10

*Note:* Pain intensity (NRS0‐10), CRPS severity score (0–16), Neuropathic Pain Symptom Inventory (0–100, zero to maximum symptom expression), pain duration in years, Disability of Hand Shoulder and Arm questionnaire (0–100, zero to maximum disability), von Korff or Graded Chronic Pain Scale (0–4, zero to maximum pain related disability), depression and anxiety percentage positive according to BDI and STA‐I results. To the first six items we could add standard deviation.

### 3.2 Cluster Switch Over the Course and Treatment of the Disease

In the subgroup of patients who participated in the follow‐up examination approximately 2.5 years later, the overall distribution pattern of CRPS patients across the NPSI clusters was largely comparable to that observed at baseline (Figure 2B). However, for many patients, the cluster assignment—that is, reported neuropathic symptoms—changed over the course of the disease. In particular, patients with evoked or pinpointed pain described alterations in symptomatology that resulted in a switch in cluster. A significant decrease in CSS (Wilcoxon test *z* = 2.946, *p* < 0.04) and reported as well as observed allodynia was observable (*χ* ^2^(1) = 10.563, *p* = 0.001) on average 2.5 ± 0.9 years after study inclusion in the follow‐up group (Reinhold et al. 2022). A slight but not‐significant decrease over time was evident in the mean scores of most NPSI pain items (*z* = −1.035, *p* = 0.301) (Figure 2C).

### 3.3 Non‐Significant Tendency of Pinpointed Pain Patients Toward Less Analgesic Standard Treatment

All patients received treatment in accordance with current clinical guidelines, including continuous physiotherapy and occupational therapy (Harden et al. 2022). Some individuals also took other medications that can have a positive effect on their symptoms or other pain conditions, like ketamine, baclofen, clonidine, triptans, lidocaine, dronabinol and dimethylsulfide. Among the 61 patients treated at the Wuerzburg centre with complete NPSI data available, opioids, anticonvulsants and antidepressants were the most prescribed pain medications. The analysis is based on the drugs the patients received at the time of study entrance listed in the method section. In percentages we observed more prescription of medication in the evoked pain cluster, concerning NOPAs in the deep pain cluster. However, none of the differences in drug treatment observed were significant (Figure 3).

**FIGURE 3:**
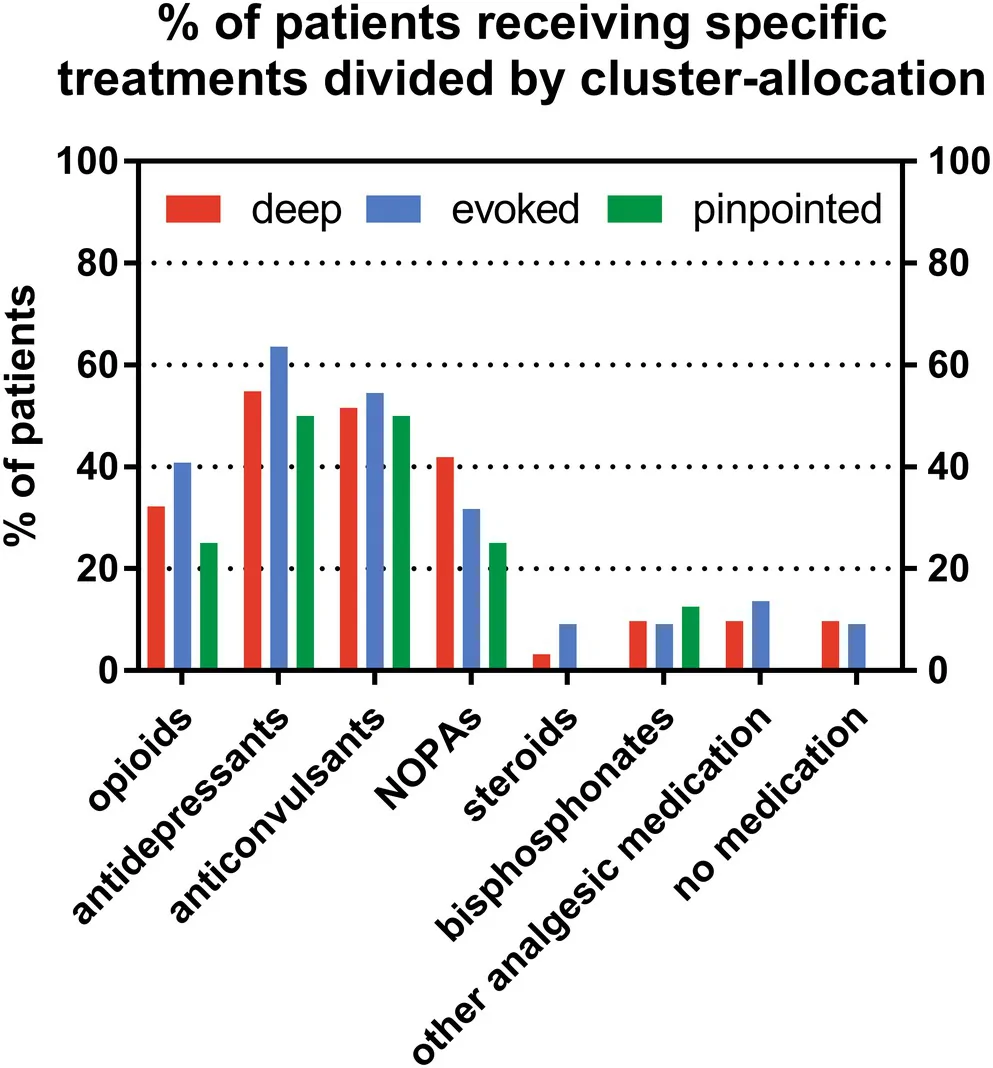
Pinpointed pain patients receive slightly less standard analgesic drugs. At the time of study inclusion, various treatment options were compared regarding their frequency for the clusters (deep: *n* = 31; evoked: *n* = 22; pinpointed: *n* = 8). ‘Other analgesic medication’ included ketamine, baclofen, clonidine, sumatriptan, local lidocaine patches, dronabinol, vitamin C and dimethylsulfide. NOPA (= non‐opioid analgesics) include non‐steroidal anti‐inflammatory drugs and metamizole. No significant differences were found, *p* > 0.05.

### 3.4 NPSI Classification Independent From Previous CRPS Subgroupings

Finally, we compared the NPSI clusters with other commonly used subgrouping approaches, including CRPS type, temperature phenotype, disease duration and gender; using Chi‐squared tests to evaluate if frequency distributions of the NPSI clusters were related to other CRPS classification methods, for example, Type I versus II or warm versus cold. Cross‐tables showed no significant associations (all *χ* ^2^(df) < 4.1, ns), except for pinpointed pain and non‐chronic CRPS, *χ* ^2^(2) = 6.05, *p* = 0.049, *φ* = 0.17.

## 4 Discussion

### 4.1 Summary

Applying the Bouhassira method, the majority of CRPS patients in our cohort were classified within the deep pain cluster. Reported neuropathic pain changed over the course of the disease—but most CRPS patients still reported deep pain after 2.5 years.

Lastly, NPSI clusters did not correlate with any of the observed common classifications for CRPS (other than a weak association of the pinpointed cluster with chronicity of CRPS) as well as clinical or sensory parameters, and could therefore represent a novel and largely independent clinical approach to CRPS patient stratification. Conversely, it is also possible that NPSI clusters capture only a limited aspect of the complex and multifactorial nature of CRPS.

### 4.2 Reduced Neuropathic Pain Characteristics in the Deep Pain Cluster With Prominent CRPS Patient Allocation

Phenotype‐based pain classifications offer several advantages in the study and management of complex pain conditions such as CRPS. Notably, they are non‐invasive and can be used internationally without the need for special equipment. One well‐established example of a successful phenotype‐based approach is the headache classification, which forms the foundation for both research and therapeutic decision‐making in this field (‘Headache Classification Committee of the International Headache Society (IHS), The International Classification of Headache Disorders, 3rd edition’ 2018). This highlights the potential value of symptom‐oriented categorization strategies for other complex pain conditions such as CRPS. In contrast, the known classification methods for CRPS, while valuable for diagnostic purposes, appear to have reached their limits when applied to the development of individualized treatment strategies (Goebel et al. 2021; Knudsen et al. 2023). We found that the NPSI clusters represent a novel method that is largely independent of traditional CRPS subgrouping factors, while potentially providing valuable insights into pain pathophysiology, particularly with regard to distinct neuropathic pain phenotypes. This non‐invasive strategy may therefore contribute to a more nuanced understanding of pain mechanisms in CRPS and support patient stratification, subgrouping and treatment optimization in both clinical practice and research settings. However, it is important to acknowledge that relying solely on NPSI‐based stratification places a strong emphasis on neuropathic and nociplastic pain dimensions and consequently overlooks other key components of CRPS, including vasomotor, sudomotor, trophic and motor manifestations.

When interpreting findings based on the NPSI clusters, it is essential to consider the stability of cluster assignment over time. The deep pain cluster seems to be the most prominent one, with many patients transitioning into or remaining within this cluster during disease progression. Contrary to initial expectations, pain scores associated with this cluster decreased over time.

This trend, together with the observed reduction in CRPS Severity Score (CSS) and decreased frequency of allodynia, suggests that the shift toward the deep pain cluster over an average of 2.5 years may reflect changes in neuropathic characteristics rather than disease worsening. Although complete recovery remains rare, these findings provide evidence for partial clinical improvement over time in a subset of CRPS patients (Reinhold et al. 2022).

To identify the potential predictors of treatment response, we specifically examined whether therapeutic strategies recommended by the German Society for Neurology (DGN) differed across NPSI‐defined pain clusters. The DGN (https://register.awmf.org/de/leitlinien/detail/030‐116) has published comprehensive guidelines on the diagnosis and treatment of CRPS, outlining current recommended treatment strategies for this condition (DGN 2018). Differences in pharmacological treatment patterns were observed according to cluster allocation using the Bouhassira algorithm. Notably, patients classified within the pinpointed pain cluster were less frequently prescribed opioids, antidepressants or non‐opioid analgesics. This trend may indicate a reduced need for, or diminished effectiveness of, these medications in CRPS patients with predominant pinpointed pain characteristics. However, these differences did not reach statistical significance and should therefore be interpreted with caution. It would be advantageous to verify this trend in future studies with a larger sample size.

Bouhassira et al. (2021) reported that patients with neuropathic pain classified within the deep and evoked pain clusters showed a more favourable response to treatment with subcutaneous Botulinum toxin A (BTX‐A) injections. BTX‐A is known to inhibit synaptic vesicle fusion with the presynaptic membrane by cleaving the synaptosome‐associated protein 25 (SNAP‐25). The resulting suppression of neurotransmitter release modulates molecular signalling mechanisms within sympathetic ganglia, sensory neurons and neuromuscular junctions (Matak et al. 2019).

Furthermore, Spagna and Attal (2023) reviewed the efficacy of BTX‐A in various neuropathic pain conditions and reported a generally favourable safety profile in combination with the most pronounced treatment responses observed in patients with evoked and deep pain with pain paroxysms. Although subcutaneous BTX‐A is not currently recommended as a standard treatment for CRPS, it represents a valuable investigational option given the neuropathic and sympathetic components of CRPS‐related pain. It is one of the treatment options for muscle spasms or dystonia, which we repeatedly see in CRPS (Siongco et al. 2020).

Clustering CRPS patients using the Bouhassira algorithm could offer a clinically meaningful stratification method to anticipate treatment response and to identify patients who may particularly benefit from targeted interventions such as BTX‐A.

### 4.3 Limitations

This study is a secondary analysis of the ncRNAPain study; therefore, the findings should be validated in an independent, prospectively designed cohort to increase robustness and generalizability. Additionally, follow‐up data were available for only a limited subset of patients (*n* = 34), restricting the statistical power of longitudinal analyses. Because symptom assessment relied primarily on self‐reported questionnaires, the results are inherently subject to reporting bias and may be influenced by patients' recall accuracy.

Furthermore, stratifying CRPS patients exclusively according to neuropathic pain symptom profiles may overlook other clinically relevant disease dimensions, including vasomotor, sudomotor and motor/trophic manifestations that are central to CRPS pathophysiology.

Even though the treatment of all CRPS patients broadly aligned with the recommendations of the DGN (DGN 2018), therapeutic interventions were not standardized across patients, limiting comparability between treatment groups.

Finally, treatment patterns were analysed retrospectively, and this study did not assess treatment efficacy or outcomes in direct association with NPSI cluster assignment.

As the three clusters of CRPS patients do not significantly differ in the investigated variables, these clusters may represent an epiphenomenon without significant clinical relevance, particularly in the absence of data on treatment response.

## 5 Conclusions

The application of the NPSI‐based clustering approach proposed by Bouhassira et al. enabled the classification of CRPS patients into distinct subgroups based on neuropathic pain symptom profiles. Cluster assignment was mostly independent of established CRPS classification schemes and may therefore serve as a complementary framework to (i) enhance the understanding of pathophysiological mechanisms underlying CRPS subtypes and (ii) improve patient stratification in clinical trials.

In cases of refractory CRPS following failure of guideline‐based therapies, BTX‐A administration in specialized centres may therefore represent a promising alternative treatment option. Ultimately, NPSI‐based clustering may facilitate a shift from purely symptom‐based management toward mechanism‐based therapeutic strategies in CRPS.

## Author Contributions

This study was designed by F.B. and H.L.R. The experiments were performed by A.‐K.R., F.B. and H.L.R. The data were analysed by G.K. and B.R.B., and the results were critically examined by all authors. B.R.B. had a primary role in preparing the manuscript, which was edited by G.K., H.L.R., A.‐K.R. and F.B. All authors have approved the final version of the manuscript and agree to be accountable for all aspects of the work.

## Funding

This work was funded by the European Commission (#602133, ncRNAPain), the German Research Foundation (KFO5001).

## Conflicts of Interest

G. Kindl, A.‐K. Reinhold and B. Rogalla von Bieberstein declares no conflicts of interest. H. L. Rittner and F. Birklein received EU funds for ncRNAPain Study (FP7).

## Supporting information


**Table S1:** Results separated by CRPS Type 1 and 2: Patients characteristics sorted by CRPS type. Significance level: *p < 0.05; **p < 0.01; non‐parametric Mann–Whitney test.
