# Supplementary material for: Classification of Complex Regional Pain Syndrome Patients Using Neuropathic Pain Symptoms—A Secondary, Retrospective Analysis From ncRNAPain
Source: Eur J Pain. 2026 Nov 6;30(10):e70425. doi: 10.1002/ejp.70425 (PMC13741284; doi:10.1002/ejp.70425)
Supplement: Supplementary file 1 [file EJP-30-0-s001.docx]

| **Table 3** | **CRPS Type 1**  **N=183** | **CRPS Type 2 N=29** |
| --- | --- | --- |
| Pain intensity ±  standard deviation | 4.9 ± 2.3 | 4.9 ± 1.9 |
| CRPS Severity Score ± standard deviation | 10.6 ± 3 | 10.8 ± 3.1 |
| NPSI sum score ± standard deviation | 36.5 ± 23* | 46.2 ± 22.2* |
| Duration of pain [years] ± standard deviation | 1.2 ± 1.9** | 2.3 ± 2.3** |
| DASH Score [0-100] ± standard deviation | 50.5 ± 23.8 | 54.4 ± 18.9 |
| GCPS Score [0-4] ± standard deviation | 3.1 ± 1.1 | 3.2 ± 1.1 |
| Depression BDI [%] | 26.7 | 44.8 |
| Anxiety STA-I [%] | 8.7 | 10.3 |
| Deep pain cluster [%] | 56.3 | 51.7 |
| Evoked pain cluster [%] | 29.5 | 34.5 |
| Pinpointed pain cluster [%] | 14.2 | 13.8 |
